# Epidemiological characteristics and spatial−temporal analysis of COVID-19 in Shandong Province, China

**DOI:** 10.1017/S095026882000151X

**Published:** 2020-07-06

**Authors:** C. Qi, Y. C. Zhu, C. Y. Li, Y. C. Hu, L. L. Liu, D. D. Zhang, X. Wang, K. L. She, Y. Jia, T. X. Liu, X. J. Li

**Affiliations:** 1 Department of Biostatistics, School of Public Health, Cheeloo College of Medicine, Shandong University, Jinan, Shandong 250012, China; 2 School of Public Health, Cheeloo College of Medicine, Shandong University, Jinan, Shandong 250012, China

**Keywords:** Cluster transmission, COVID-19, epidemiological characteristics, spatial−temporal analysis

## Abstract

The pandemic of coronavirus disease 2019 (COVID-19) has posed serious challenges. It is vitally important to further clarify the epidemiological characteristics of the COVID-19 outbreak for future study and prevention and control measures. Epidemiological characteristics and spatial−temporal analysis were performed based on COVID-19 cases from 21 January 2020 to 1 March 2020 in Shandong Province, and close contacts were traced to construct transmission chains. A total of 758 laboratory-confirmed cases were reported in Shandong. The sex ratio was 1.27: 1 (M: F) and the median age was 42 (interquartile range: 32–55). The high-risk clusters were identified in the central, eastern and southern regions of Shandong from 25 January 2020 to 10 February 2020. We rebuilt 54 transmission chains involving 209 cases, of which 52.2% were family clusters, and three widespread infection chains were elaborated, occurring in Jining, Zaozhuang and Liaocheng, respectively. The geographical and temporal disparity may alert public health agencies to implement specific measures in regions with different risk, and should attach importance on how to avoid household and community transmission.

## Introduction

Coronavirus disease 2019 (COVID-19), which was identified in early December 2019, brought out the international concerns of the public health impact of a new infectious disease outbreak [1–5]. The pandemic of COVID-19 led to severe damages all over the world, causing a strong impact on the economy and health systems [6].

To explore the characteristics of COVID-19 and support the prevention and control strategy, the epidemiology of COVID-19 has been studied with the development of the disease [7–11]. Nevertheless, there remain many knowledge gaps in the study of COVID-19 [12, 13]. The spatial−temporal heterogeneity of COVID-19 outbreak was not yet explored, as it could be instrumental to scientific evidence-based policy implementation. Also, it has been reported that COVID-19 could be effectively transmitted inside the family and community [14–16], and the transmission in the community could largely impact on the epidemic trajectory [17]. While the details in cluster transmission were not clear enough, further study for person-to-person transmission in households and other locations would be valuable [15].

In this study, we provided an analysis of data from laboratory-confirmed cases in Shandong Province to describe the epidemiological characteristics and transmission chains of COVID-19 and explain the details of transmission between close contacts.

## Methods

### Data collection

Data on laboratory-confirmed COVID-19 cases in Shandong Province, China, between 21 January 2020 and 1 March 2020 were obtained from Provincial and Municipal Health Commissions. We collected information about regions (cities, districts or counties), sex, age, date of symptom onset, confirmed date and close contact. Population size for each city, district and age group was obtained from the published Shandong Statistical Yearbook in 2019 and the Sixth Census in Shandong Province from Shandong Province Bureau of Statistics, respectively.

### Study area

**Fig. 1.**
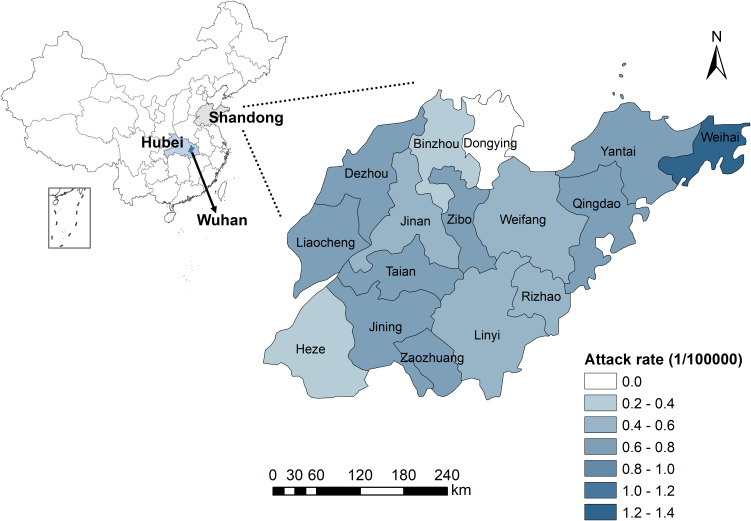
The spatial distribution of attack rate (per 100 000 population) of 558 laboratory-confirmed COVID-19 cases, Shandong Province, China. Dongying has no confirmed case, and the city-level attack rate varied from 0.21 per 100 000 to 1.34 per 100 000.

Shandong Province, which is located in eastern China (Fig. 1), has 16 cities and 137 counties or districts. Shandong has a monsoon climate with an area of 157 965 km^2^ and a total population of 100 472 400 at the beginning of 2019.

### Statistical analysis

The attack rate of COVID-19 was calculated and the spatial distribution was mapped for 16 cities in Shandong. The median time from symptom onset to diagnosis was calculated based on the available data. We constructed the epidemic curve of confirmed date with public health intervention measures. For county-level analysis, spatial and space−time scan analysis were performed to detect any spatial−temporal cluster in which the number of cases within the cluster was significantly higher than the expected number of cases in the rest of the region. In this analysis, we used a Poisson model with the maximum spatial cluster size set at less than 30% of the total population and maximum temporal cluster size set at 50% of the total study period to scan possible sub-clusters. Based on the detailed close contacts data, we constructed transmission chains for clusters of cases with known contacts.

Data were analysed using R 3.6.0. The spatial−temporal scan analysis was conducted by SaTScan v9.6. The visualisation of maps was mapped using the geographical information system (GIS) technique in ArcGIS 10.5 software (ESRI Inc., Redlands, CA, USA).

## Results

### Descriptive analysis

As of 1 March 2020, a cumulative total of 758 laboratory-confirmed COVID-19 cases were reported in Shandong Province, China (200 cases reported in a special group on 20 February 2020, disconnected from the outside world and cannot obtain detailed information, which was excluded from the study). Considering the confirmed cases who had both dates of symptom onset and diagnosis, we calculated the median time from onset to diagnosis was 5 days (range 0–16 days; interquartile range (IQR): 2–8 days). Among the reported cases (eight cases were removed with missing values of sex and age), the sex ratio was 1.27: 1 (M: F), and the median age was 42 (range 9 months - 91 years old; IQR: 32–55). The age distribution of cases and age-specific attack rates at the city level are shown in Supplementary Figure S1. The attack rates in the age groups of more than 85 years old were higher than other groups. For instance, the attack rate of 90–94 age group was 9.14 per 100 000 in Jining and 8.02 per 100 000 for 85–89 age group in Taian. The spatial distribution of attack rate is shown in Figure 1. The city-level attack rate varied from 1.34 per 100 000 in Weihai to 0.21 per 100 000 in Heze.

**Fig. 2.**
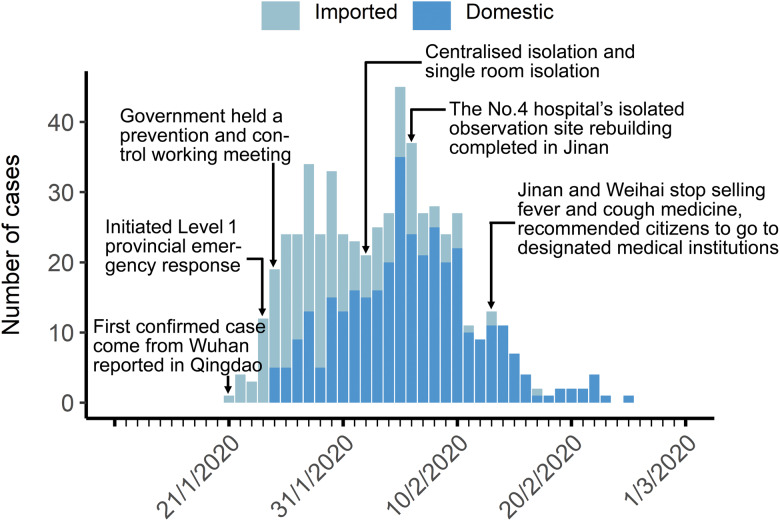
Epidemic curves of confirmed date as of 1 March 2020 for confirmed COVID-19 cases for Shandong Province, China. First stage: 21–24 January 2020; second stage: 25 January−10 February 2020; third stage: 11 February–1 March 2020.

The epidemic curve of confirmed cases is illustrated in Figure 2, representing three stages in the outbreak process. The daily confirmed cases were less than 20 in the first stage with most cases were imported cases who came from outside the province. The second stage began on 25 January with more than 20 cases reported per day, and the number of domestic cases increased with each passing day. The high prevalence has continued for about 16 days in Shandong. After 10 February, the number of newly confirmed cases was decreased day by day in the third stage.

### Spatial−temporal analysis

**Fig. 3.**
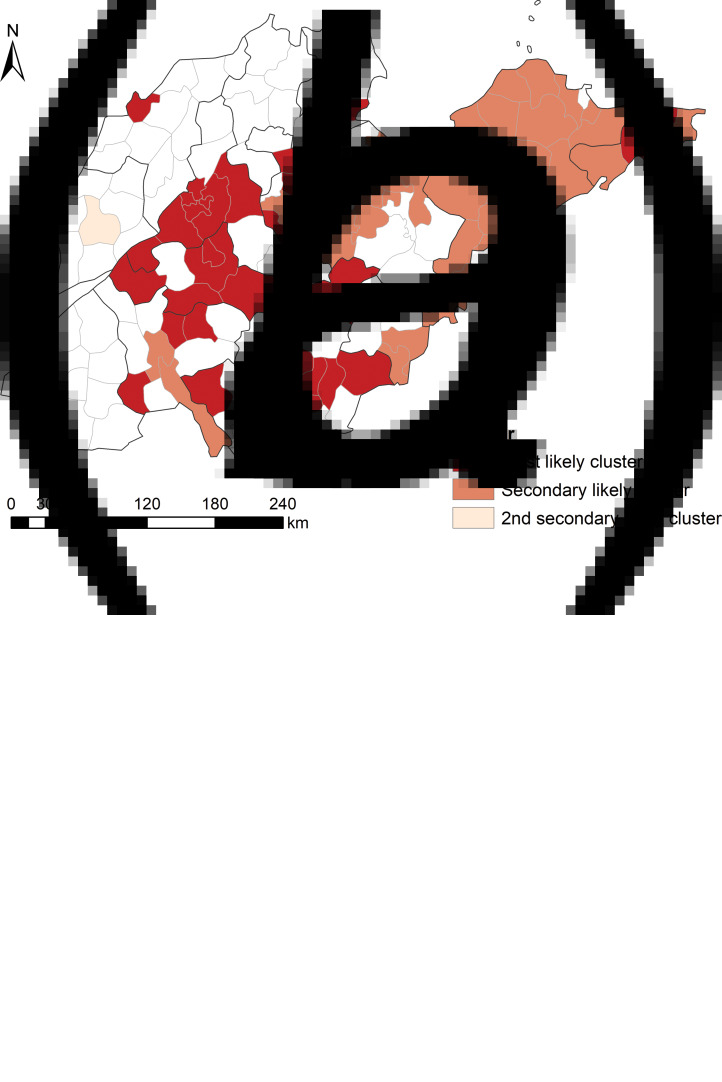
Spatial clusters (a) and space−time clusters (b) of COVID-19 confirmed cases with significant higher risk in Shandong Province, China, from 21 January to 1 March 2020.

Spatial scan analysis of COVID-19 cases in Shandong Province identified one most likely cluster and three secondary clusters (Fig. 3a and Supplementary Table S1). Spatial clusters in 137 counties showed that the high-risk regions are in central and eastern Shandong. The relative risk (RR) within the most likely cluster was 2.11 (*P* < 0.0001), with 138 observed cases compared with 75.07 expected cases. The RR of secondary clusters was also significant (*P* < 0.001).

The space−time scan analysis identified the high-risk clusters in the central, eastern and southern regions of Shandong from 25 January 2020 to 10 February 2020 (Fig. 3b). The RR within the most likely cluster was 3.99 (*P* < 0.0001) with 180 observed cases compared with 59.51 expected cases, and the RR of secondary clusters was also significant (*P* < 0.0001). The significant time frame and cluster areas of high risk are listed in Table 1.

**Table 1.** Space−time clusters with significant higher risk in Shandong Province, China, from 21 January 2020 to 1 March 2020

Cluster	Time frame	Location (county or district)	Cases	Expected	RR	*P*-value
Most likely	25 January 2020–10 February 2020	Huancui, Yanzhou, Luozhuang, Lanshan (Linyi), Decheng, Dongping, Mengyin, Ningyang, Taishan, Shizhong, Linzi, Xintai, Jvnan, Daiyue, Yishui, Jinxiang, Wendeng, Zhangdian, Changqing, Qufu, Licheng, Shizhong, Hengtai, Lixia, Tengzhou, Feixian, Dongying, Zhangqiu, Huaiyin, Hedong, Pingyin, Boshan, Tianqiao	180	59.51	3.99	<0.0001
Secondary	24 January 2020–10 February 2020	Laishan, Longkou, Fushan, Muping, Weicheng, Lanshan (Rizhao), Hanting, Rushan, Rongcheng, Qixia, Penglai, Laizhou, Donggang, Zichuan, Haiyang, Laiyang, Pingdu, Zhaoyuan, Laixi, Jiaozhou, Licang, Chengyang, Huangdao, Shibei, Jimo, Weishan, Rencheng, Linqu, Laoshan, Kuiwen, Changyi, Changle	154	53.82	3.57	<0.0001
2^nd^ secondary	4 February 2020–8 February 2020	Dongchangfu	22	0.87	26.42	<0.0001

RR, Relative risk.

### Transmission chains analysis

**Fig. 4.**
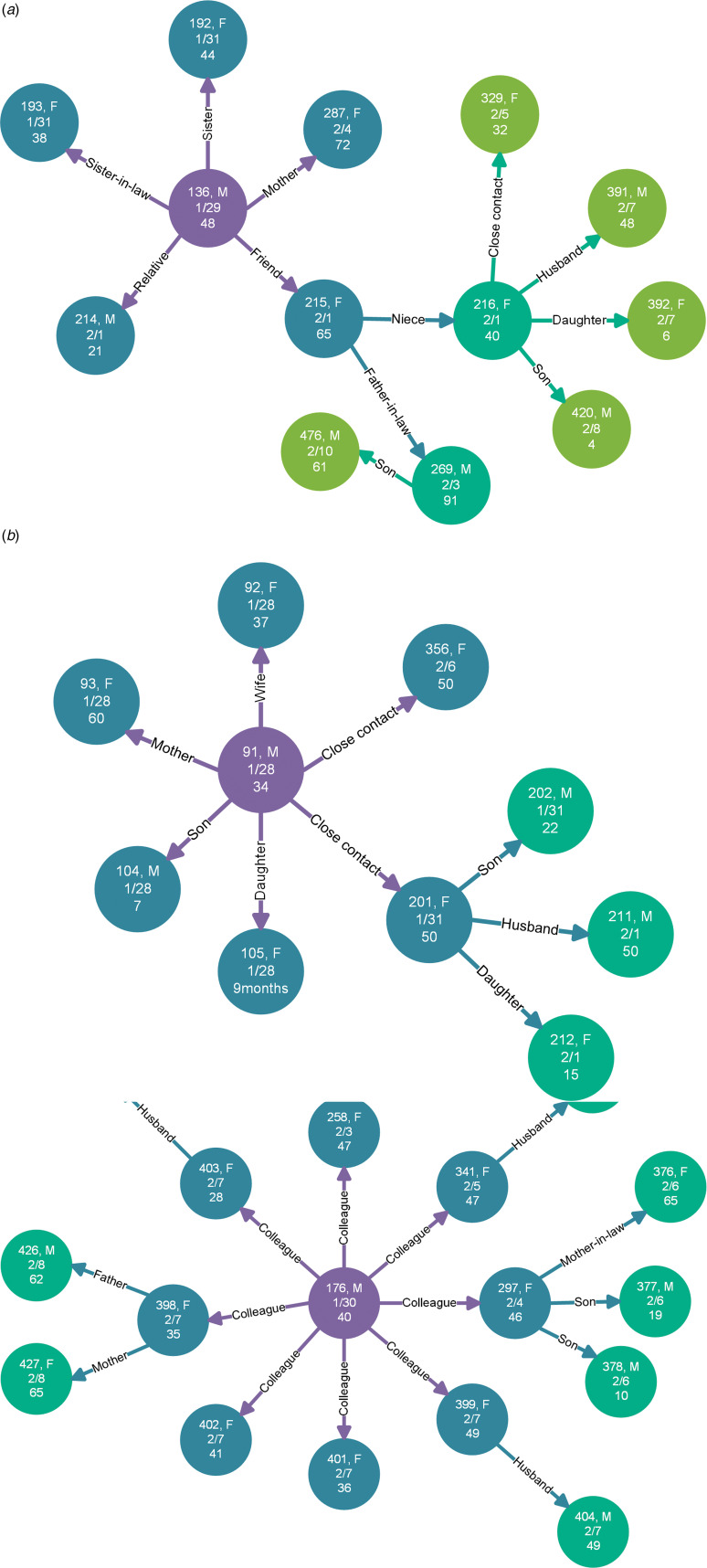
Transmission chains of some clusters in Shandong Province, China. Circles represent individual cases, and edges show the epidemiological contacts reported between them. Arrows represent the direction of transmission, and the words on the edges display the relationship of the infected case to the infector, as well as the colour of nodes corresponds to the infection order of cases. Violet, cyan, blue and green represent the first generation, second generation, third generation and fourth generation, respectively. Information in nodes show case number, sex (F/M: female or male), confirmed date and age. (a) Cluster in Qufu city, Jining city; (b) cluster in Shizhong district, Zaozhuang city; (c) cluster started in a supermarket in Dongchangfu district, Liaocheng city.

The investigation of clustering infection showed that, among a total of 209 cases reported in clusters, 54 transmission chains, involving 182 infected cases, occurred in Shandong Province (Supplementary Fig. S2). Also, most cases were household transmissions that account for 52.2% of cases reported in clusters. We structured three transmission chains that caused widespread infection and presented the information including case number, sex, age and confirmed date (Fig. 4). The family and community cluster occurred in Jining, has the highest number of cases. The cluster infected to the fourth-generation cases (Fig. 4a). Clustering infections occurred in Zaozhuang and Liaocheng were passed to the third-generation cases. The cluster of Zaozhuang (Fig. 4b) was similar to Jining, which mainly occurred in family and community. However, a large supermarket clustering infection has occurred in Liaocheng, with 17 contacts linked to a supermarket called Zhenhua, located in Dongchangfu District (Fig. 4c). However, nine cases were staff members of the supermarket, and the other eight cases were family members of four workers.

## Discussion

No newly confirmed case has been reported for more than 10 days after the last study date (1 March 2020), indicating that the collected data covered a virtually complete outbreak process in Shandong Province.

In the 550 laboratory-confirmed cases, the sex ratio was 1.27: 1, which is similar to the result based on the first 425 cases in Wuhan reported by Li *et al*. [15]. Females have reduced susceptibility probably because of the function of sex hormones, which play a part in innate and adaptive immunity [18, 19]. The median age of cases was 42 (IQR: 32–55), whereas a previous study reported that most cases were aged 30–69 (77.8%) on the basis of 44 672 confirmed cases of COVID-19 diagnosed nationwide in China by the end of 11 February 2020 [20]. The high attack rates in the age groups of more than 85 years old indicated that elder people with weaker immune function were more likely to be infected [18].

The COVID-19 epidemic in Weihai City was serious, displayed in Figure 1, probably because the population size of Weihai is smaller than other cities and the attack rate is a relative number, so the attack rate was higher than others. Besides, Weihai is one of the major migration cities in Shandong. As a tourist city, it attracts many visitors, causing a higher probability for infection. Dongying city did not have any confirmed cases. There are several possible explanations: first, Dongying, as a city with the lowest population density of 263 per km^2^, has a low risk of transmission of diseases, while the average population density of Shandong is 636 per km^2^ [21]. Second, Dongying is not highly mobile, with fewer migrant workers due to its underdeveloped economy. Third, there is no high-speed rail, which is the preferred way to travel in China, so it has a lower opportunity of imported cases. Moreover, some people who returned from epidemic areas were not infected with COVID-19.

COVID-19 was incorporated into the statutory reporting of infectious diseases on 20 January 2020, and the management of Class B infectious diseases and measures for Class A infectious diseases were adopted [22]. The first imported case, coming from Wuhan to Shandong, was confirmed in Qingdao on 21 January 2020 (Fig. 2). The first confirmed cases in each city were all imported cases from Wuhan or other regions (Supplementary Table S2). Due to Wuhan's position as a transportation hub and the high mobility of the population during the Chinese Lunar New Year travel season, infected people have moved into all parts of China, especially cities frequently communicated with Wuhan. All imported cases left Wuhan before 23 January 2020, after which, Wuhan implemented strict traffic restrictions [23]. The highest provincial emergency response to COVID-19 issued by Shandong provincial government was initiated on 24 January 2020. The plans for case isolation and treatment were improved. Measures were implemented to ensure all cases were diagnosed and treated, and close contacts were traced and isolated under medical observation. The time before 24 January was considered as the first stage, when the cases were all imported from outside the province. The period from 25 January 2020 to 10 February 2020 was the second stage of the epidemic curve and it was the period of high prevalence, which is consistent with the time frame of high risk identified by space−time scan analysis.

At the national level, the Chinese Lunar New Year holiday in 2020 was extended to reduce the transportation capacity and control the transmission of COVID-19 [24]. Public health control measures like social distancing, closure of schools (universities and public places), mandatory quarantine and daily reporting have been implemented [25, 26]. For the public, facemasks and hand hygiene were effective to prevent person-to-person spread of the disease [24, 27]. In the later stage, a rapid decrease has been seen in China, and students and workers were arranged to return schools and institutions in phases and batches.

Spatial and space−time scan analysis aimed to detect the aggregation of COVID-19 cases and test any statistically significant clusters. The spatial scan analysis is useful for COVID-19 analysis of cluster patterns. The space−time scan analysis is a dynamic detection for examining the spatial−temporal variation of risk [28]. The areas and time frames with COVID-19 had heterogeneity characteristics in Shandong Province from 21 January 2020 to 1 March 2020. Except high-risk areas the same as spatial scan, the most likely cluster in southern Shandong may be caused by vendors who sold fried sunflower seeds in Wuhan. They went back to Linyi city since the Chinese Lunar New Year. However, even the number of imported cases in Linyi was the largest one in Shandong Province (Supplementary Table S3), the local cases were less than most cities in Shandong, indicating that there were effective prevention and control measures in Linyi.

Transmission chains surveys can help in describing household and community infections [13, 29]. The cluster epidemic was significant in Jining, where cluster infections spread to fourth-generation cases, which may be because the clinical symptoms of the first-generation cases were not obvious, as well as relatives of these cases did not have enough awareness for prevention [30]. The index case numbered 136 had come from Beijing (Fig. 4a), and was infected during travel. The index case numbered 91 (Fig. 4b) came from Wuhan was also an imported case. Whereas, the index case numbered 176 (Fig. 4c) was not imported. He may be infected while working in the supermarket in Liaocheng. The cluster in the supermarket was caused by underreporting and concealment. If timely diagnosis and isolation measures were implemented, the cluster should have been avoided. These findings indicated that transmission-reduction interventions will be beneficial to mitigate infection and involve citizens in active prevention [4, 26].

In conclusion, this study provides the epidemiological characteristics of COVID-19 in Shandong Province, China. The geographical and temporal disparity indicates that specific risk-reduction programmes should be formulated for regions with different risks. High-risk areas should be aimed to strengthen prevention and control measures, and further studies should be conducted to conclude the specific risk factors. The cluster transmission in the study implies that public health institutions should pay more attention to family and community clusters. The spread of the epidemic has been contained effectively in China. However, the situation remains serious worldwide. This study may provide a reference for future studies on the COVID-19 epidemic.

## Data Availability

The data of this study are available from official website of the Provincial and Municipal Health Commissions in Shandong.

## Supplementary material

For supplementary material accompanying this paper visit http://dx.doi.org/10.1017/S095026882000151X. click here to view supplementary material
