# Supplementary material for: Epidemiological characteristics and spatial−temporal analysis of COVID-19 in Shandong Province, China
Source: Epidemiol Infect. 2020 Jul 6;148:e141. doi: 10.1017/S095026882000151X (PMC7360956; doi:10.1017/S095026882000151X)
Supplement: Supplementary file 1 [file S095026882000151Xsup001.docx]

***Epidemiology and Infection***

**Epidemiological characteristics and spatial-temporal analysis of COVID-19 in Shandong Province, China**

C. Qi,^1^ Y. C. Zhu,^1^ C. Y. Li,^1^ Y. C. Hu,^2^ L. L. Liu,^1^ D. D. Zhang,^1^ X. Wang,^1^ K. L. She,^1^ Y. Jia,^1^ T. X. Liu,^1^ X. J. Li^1^

^1^Department of Biostatistics, School of Public Health, Cheeloo College of Medicine, Shandong University, Jinan, Shandong 250012, China, ^2^School of Public Health, Cheeloo College of Medicine, Shandong University, Jinan, Shandong 250012, China

**Supplementary Material**

A**
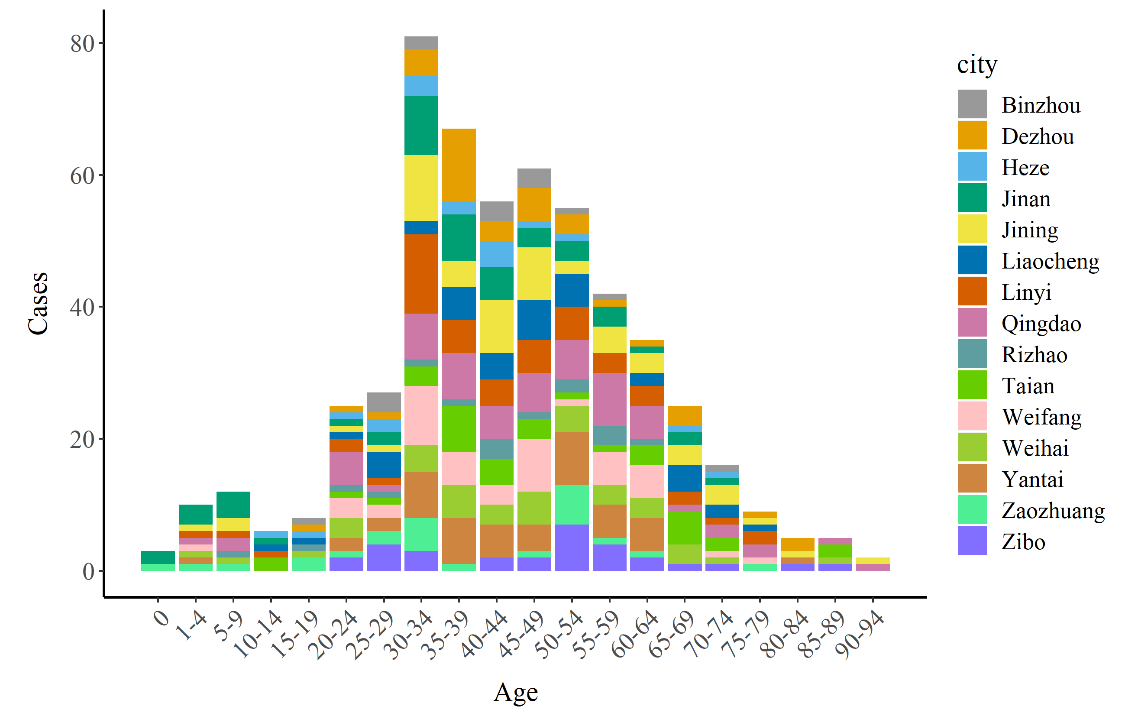
**

B
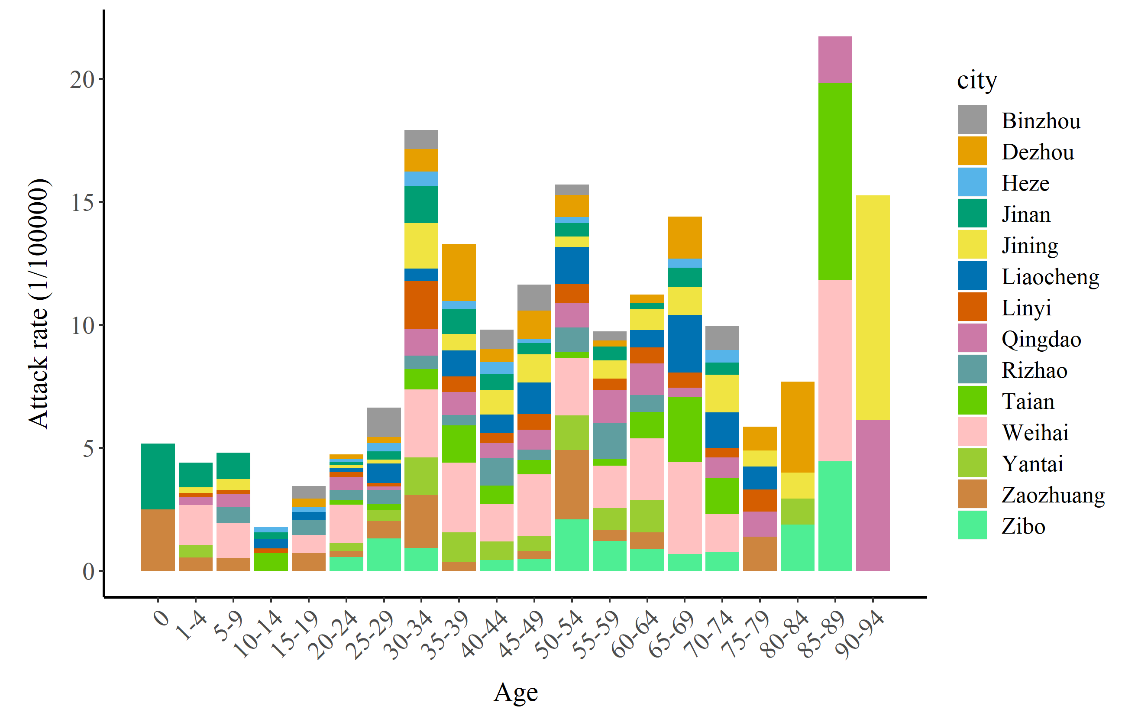


Figure S1. Age distribution of COVID-19 cases and attack rate in 15 cities (except Dongying), in Shandong Province, China. (A) Cases in the city of Shandong; (B) Attack rate in the city of Shandong.


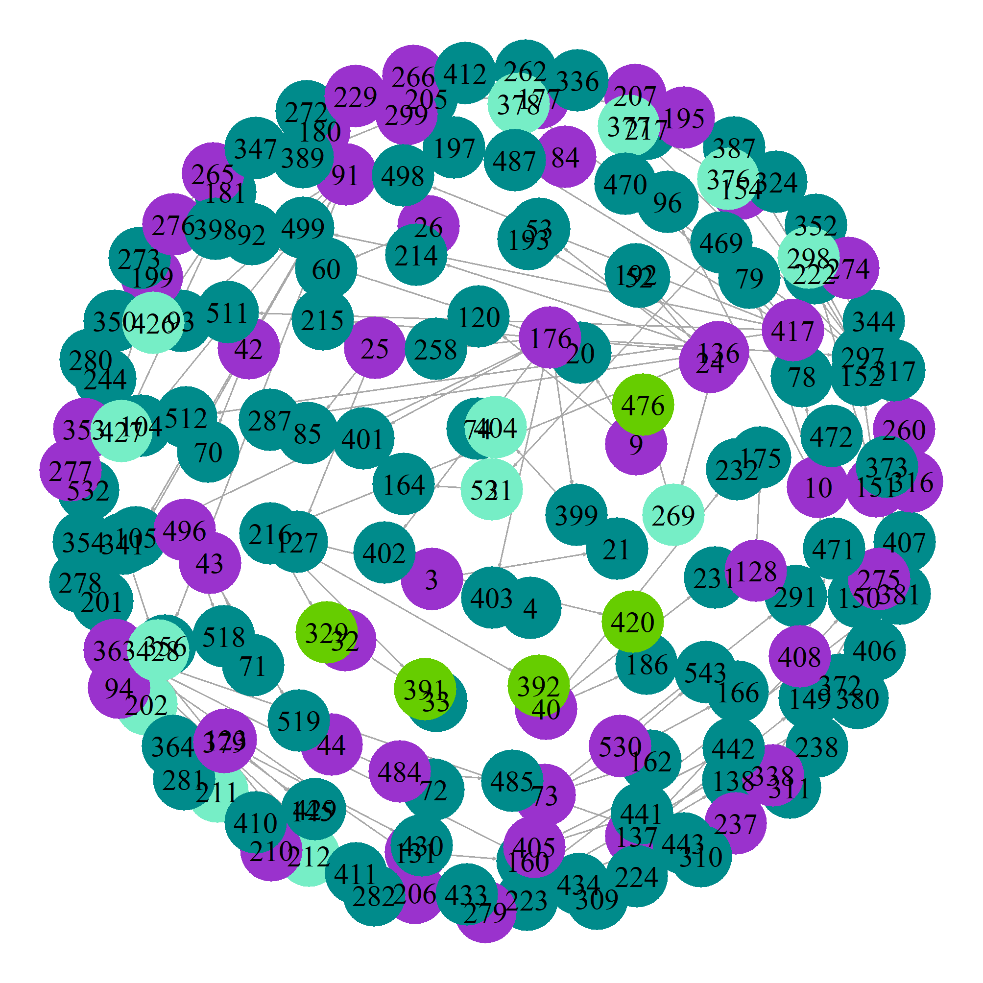


Figure S2. Transmission net of 182 cases of COVID-19 in Shandong Province, China. Circles show individual cases, and edges represent transmission relationship between them. Color of nodes corresponds to the infection generation of cases. Violet, cyan, blue and green represent the first generation, second generation, third generation and forth generation respectively. The transmission net was constructed by “igraph” package in R 3.6.0.

Table S1. Spatial clusters with significant higher risk in Shandong Province, China

| Cluster | Location (county or district) | Cases | Expected | RR^*^ | *P*-value |
| --- | --- | --- | --- | --- | --- |
| Most likely | Ningyang, Dongping, Changqing, Wendeng, Shizhong, Licheng, Lixia, Xintai, Huaiyin, Huancui, Lanshan, Pingyin, Luozhuang, Tianqiao, Taishan, Zhangqiu, Yanzhou, Qufu | 138 | 75.07 | 2.11 | <0.0001 |
| Secondary | Fushan, Longkou, Muping, Laishan, Weicheng, Qixia, Laizhou, Lanshan, Rongcheng, Hanting, Rushan, Penglai, Laiyang, Haiyang, Donggang, Zichuan, Zhaoyuan, Pingdu, Laixi, Jiaozhou, Chengyang, Licang, Huangdao, Shibei, Weishan, Jimo, Rencheng, Kuiwen | 180 | 108.98 | 1.96 | <0.0001 |
| 3^th^  secondary | Dongchangfu | 26 | 7.10 | 3.79 | <0.0001 |
| 4^th^  secondary | Wucheng | 13 | 2.23 | 5.95 | 0.0003 |

^*^ RR: Relative risk.

Table S2. The first confirmed case of COVID-19 for 16 cities in Shandong Province, China

| City | Gender | Age | Hospitalized | Confirmed date | Come from |
| --- | --- | --- | --- | --- | --- |
| Jinan | Female | 29 | 1/23/2020 | 1/24/2020 | Wuhan |
| Qingdao | Male | 37 | 1/17/2020 | 1/21/2020 | Wuhan |
| Zibo | Male | 26 | 1/22/2020 | 1/25/2020 | Nanjing |
| Zaozhuang | Female | 51 | 1/25/2020 | 1/27/2020 | Thailand |
| Yantai | Female | 56 | 1/23/2020 | 1/24/2020 | Wuhan |
| Weifang | Female | 62 | / | 1/23/2020 | Wuhan |
| Jining | Female | 57 | 1/21/2020 | 1/24/2020 | Wuhan |
| Taian | Male | 31 | 1/24/2020 | 1/27/2020 | Wuhan |
| Weihai | Male | 38 | 1/20/2020 | 1/22/2020 | Zhejiang |
| Rizhao | Male | 25 | 1/22/2020 | 1/24/2020 | Wuhan |
| Linyi | Male | 65 | 1/21/2020 | 1/22/2020 | Hubei |
| Dezhou | Male | 35 | 1/23/2020 | 1/24/2020 | Wuhan |
| Liaocheng | Female | 28 | 1/23/2020 | 1/24/2020 | Wuhan |
| Binzhou | Female | 59 | 1/24/2020 | 1/25/2020 | Wuhan |
| Heze | Male | 26 | 1/20/2020 | 1/25/2020 | Wuhan |
| Dongying | No case | | | | |

Table S3. The number of imported and domestic confirmed cases of COVID-19 for each city in Shandong Province, China

| Imported | |  | Domestic | |
| --- | --- | --- | --- | --- |
| City | Number of cases |  | City | Number of cases |
| Linyi | 29 |  | Jining | 50^*^ |
| Jinan | 25 |  | Qingdao | 36 |
| Qingdao | 23 |  | Yantai | 31 |
| Weihai | 19 |  | Liaocheng | 29 |
| Yantai | 17 |  | Weifang | 29 |
| Weifang | 14 |  | Dezhou | 24 |
| Dezhou | 13 |  | Taian | 23 |
| Taian | 12 |  | Jinan | 22 |
| Heze | 11 |  | Linyi | 20 |
| Jining | 10 |  | Zibo | 20 |
| Zibo | 10 |  | Weihai | 19 |
| Binzhou | 9 |  | Zaozhuang | 19 |
| Liaocheng | 9 |  | Rizhao | 12 |
| Rizhao | 5 |  | Heze | 7 |
| Zaozhuang | 5 |  | Binzhou | 6 |

^*^ Excluded 200 cases occurred in a special group.
